# Host-dependent morphology of *Isthmiophora melis* (Schrank, 1788) Luhe, 1909 (Digenea, Echinostomatinae) – morphological variation vs. molecular stability

**DOI:** 10.1186/s13071-015-1095-8

**Published:** 2015-09-22

**Authors:** Joanna Hildebrand, Maja Adamczyk, Zdzisław Laskowski, Grzegorz Zaleśny

**Affiliations:** Department of Parasitology, Institute of Genetics and Microbiology, Wrocław University, Przybyszewskiego 63, 51-148 Wrocław, Poland; Institute of Parasitology, Polish Academy of Science, Twarda 51/55, 00-818 Warszawa, Poland; Department of Systematic and Ecology of Invertebrates, Institute of Biology, Wrocław University of Environmental and Life Sciences, Kożuchowska 5b, 51-631 Wrocław, Poland

**Keywords:** *Isthmiophora melis*, Rodents, Phenotypic plasticity, Molecular taxonomy

## Abstract

**Background:**

Echinostomes are cosmopolitan digenean parasites which infect many different warm-blooded hosts. Their classification is extremely confused; the host spectrum is wide, and morphological similarities often result in misidentification. During our long-term studies on the helminth fauna of rodents and carnivores we have collected 27 collar-spined echinostomes which differ in morphology to an extent that suggests the presence of more than one species. Here, we describe this material, and the extent of host-related variation in this parasite.

**Methods:**

Specimens of *Isthmiophora* isolated from four host species (badger, American mink, hedgehog, striped field mouse) were subject to morphological and molecular examination; the data were statistically analysed.

**Results:**

Our results show that genetically all the *Isthmiophora* specimens obtained from all the examined hosts are conspecific and represent *I. melis*. On the other hand, the individuals isolated from *Apodemus agrarius* are morphologically distinct and, based on this criterion alone, should be described as a new species.

**Conclusions:**

The morphological traits of *Isthmiophora melis* are much variable and host-dependent; without molecular analysis they would suggest a necessity to describe a new species or even genus. Such a high level of intraspecific variability may be affected by the host’s longevity.

## Background

Since molecular techniques became commonly used in taxonomic studies, the list of valid taxa in different groups of organisms has been changing, and in many cases the results of molecular investigations are radically different from those obtained with classical methods. However, while museum collections dating from the pre-molecular period remain the cornerstone of taxonomy, morphology must continue to provide a starting point for molecular studies [1]. Molecular taxonomy has also contributed to revealing the common occurrence of cryptic species in nature, in virtually all major taxa. Although such species are genetically distinct from each other, they are morphologically very similar [2, 3].

On the other hand, free-living organisms and parasites can adjust their life-history strategies and a given genotype may produce a variety of phenotypes under different environmental conditions [4]. Due to their exposure to widely differing environmental conditions (i.e. different host species, host’s immune system), parasites often display a phenotypic plasticity which is expressed as differences in body size or fecundity [4]. In the case of Digenea, most species-diagnostic features are the body proportions or the shape and location of internal organs. Phenotypic variation may be induced by differences in the intensity of infection (“crowding effect”) and in the host’s identity (“host-induced variation”) [5]. These phenotypic effects may lead to species-specific variation resulting in misidentification [6].

Echinostomes are cosmopolitan digenean parasites which mainly infect many different warm-blooded hosts [7]. The taxonomy and classification of the echinostomes is highly confused. The wide host spectrum of echinostomes is a result of phylogenetic, physiological, and ecological adjustments between the parasite and the host in a dynamic evolutionary process, where the main factor influencing the host specificity is the host’s behaviour, particularly the feeding habits of vertebrate hosts [7]. Species misidentifications have arisen because of similarities in morphology and because of the lack of isolates in molecular databases [8]. Most studies have focused on the genus *Echinostoma,* especially the “*revolutum*” group, e.g. [7–14], see Kostadinova and Gibson [11] for review. Despite numerous studies, two recent papers [15, 16] showing that even by the use of molecular tools the taxonomy of this group is still not straightforward. However, taxonomic difficulties are also known in other groups of the Echinostomatinae [17, 18]. One of the remaining interesting issues concerns the 27-collar-spined echinostomes of the genus *Isthmiophora* Luhe, 1909. The type-species, *I. melis*, is a parasite reported mainly from European, Asian and American carnivores. However, Radev et al. [19], in his review of literature, lists c. 30 species as definitive hosts of this parasite. Host-induced morphological variation within this digenean, which apparently lacks host specificity, should be clearly visible.

During our long-term studies on the helminth fauna of rodents and carnivores we have collected 27 collar-spined echinostomes which differ in morphology to an extent suggesting the presence of more than one species. Molecular studies, on the other hand, suggested that these worms belong to *Isthmiophora*. Here we describe this material, and the extent of host-related variation in this parasite.

## Methods

### Parasite sampling

Representatives (*N =* 148) of *Isthmiophora* used for the morphological analysis were collected from four host species: striped field mouse (*Apodemus agrarius*, *N =* 37), European badger (*Meles meles, N =* 13), American mink (*Neovison vison, N =* 64) and European hedgehog (*Erinaceus europaeus, N =* 34), all captured during parasitological and faunistic studies carried out by the Department of Parasitology in cooperation with the Polish Academy of Sciences. The rodents were captured in Lower Silesia (Dolina Baryczy, Nature Reserve “Stawy Milickie”, 51°31′56″N/17°20′12″E) in 2010 – permission 46/2008 issued by the Second Local Commission for Animal Experiments, worms form the mink (N. Poland; Marzęcino, 54°13′1.54″N 19°13′20.43″E) captured in 2010 were obtained from the Polish Academy of Sciences, trematodes from the hedgehog and badger (2010) were obtained from the Czech Republic (Zahlinice, 49°17′06″N/17°28′41″E). After washing in tap water, the worms were fixed in 70 % ethanol. Some of the collected trematodes were stained in iron-aceto-carmine [20], dehydrated in a graded ethanol series, cleared in clove oil, mounted in Canada balsam and identified according to Kostadinova and Gibson [17]. The voucher specimens of trematodes obtained from each hostare deposited in the polish helminthological collection of Natural History Museum of Wroclaw University (MNHW).

### Statistical analysis

All the examined specimens of *I. melis* were subject to detailed morphological and morphometric analysis, including the following measurements: body length (L), maximum body width (W), body area (BA), maximum body width as a proportion of body length (BW), forebody length (FB), forebody as a proportion of body length (FO), hindbody length (HB), hindbody as a proportion of body length (H), post-testicular region length (PTR), post-testicular region length as a proportion of body length (T), oral sucker area (OSA), ventral sucker area (VSA), anterior testis area (ATA), posterior testis area (PTA), ovary area (OA), gonad area/body area (GA/BA), ventral sucker to ovary distance as a proportion of body length (U), egg length (EL), egg width (EW). The body and gonad areas were calculated using the following equations: body area = π*(body length/2)*(body widith/2); gonads area = π*r^2^. All the measurements were expressed in micrometers and proportions as percentage. Prior to the analysis the data were log-transformed (log_10_). The mean (M), minimum/maximum values and coefficients of variation (CV %; defined as the ratio of standard deviation to the mean) were calculated for all the variables. One-way analysis of variance (ANOVA) was carried out to test if the particular morphological features of *Isthmiophora* differed between the host species. In the next step we performed discriminant analysis. To avoid the size effect of the worms (*Isthmiophora* spp. isolated from the badger was much bigger than those from the other hosts) only variables expressed as ratios (BW, FO, H, T, U, GA/BA) were included in this analysis. Moreover, according to the literature data, the major diagnostic characters in this taxon are based on ratios (i.e. BW, FO, T and U). All the analyses were conducted using Statistica 10.0 software.

### Molecular analysis

Molecular analysis was performed for *I. melis* collected from four host species studied, from which a set of two worms was used for the analysis (*N =* 8). DNA was extracted using the DNeasy Blood and Tissue Kit (Qiagen), and amplified using PCR specific for 2 nuclear markers (internal transcribed spacers 1 and 2 [ITS1, ITS2] and a fragment of mitochondrial cytochrome oxidase I [*CO1*] gene) (Table 1). Two additional molecular markers (SSU and LSU of rDNA) were amplified for the specimens from *A. agrarius*. PCR conditions included initial denaturation in 95 °C for 5 min, followed by 35 cycles: 45 s denaturation (95 °C), 30 s annealing (52 °C for SSU, LSU, ITS 1, ITS 2 and 48 °C for COI), 30 s elongation (72°), and a 5 min step of final elongation (72 °C). PCR products were sequenced using the same primer pairs, and chromatograms inspected visually for ambiguities. In order to elucidate any homologies with the previously deposited sequences in GenBank, we conducted a BLAST search (http://blast.ncbi.nlm.nih.gov/Blast.cgi?CMD = Web&PAGE_TYPE = BlastHome). Multiple alignment was done using CLUSTAL W in MEGA 5.0 package [21]. The sequences obtained in this study were deposited in GenBank under the following accession numbers: [GenBank: KT359582] and [GenBank: KT359583] for SSU and LSU; [GenBank: KT359584] for ITS complex; [GenBank: KT359580] and [GenBank: KT359581] for COI (Table 1).

**Table 1:** The list of host species used for molecular identification of *I. melis* with the Gen Bank accession numbers of newly obtained sequences

Host species	Locality	Target genes/primers reference			
		18S rDNA/ [33]	28S rDNA/ [34]	ITS rDNA/[12]	COI mtDNA/[13]
*Apodemus agrarius*	Poland	KT359582	KT359583	KT359584	KT359580
*Erinaceus europaeus*	Czech Republic				
*Nevison vison*	Poland				KT359581
*Meles meles*	Czech Republic				

## Results

### Molecular analysis

The morphological distinctness of *I. melis* from the striped field mouse did not permit unambiguous identification of the parasite to specific or even generic level. Two markers 18S rDNA (1078 bp) and 28S rDNA (1352 bp) were therefore used for preliminary identification. BLAST analysis showed 99 % similarity with the sequences of *I. melis* [GenBank: AY222131] and [GenBank: AF151941] and *I. hortensis* [GenBank: AB189982] for both loci. Amplification of *COI* from the four host species generated sequences of 222–261 bp. Two haplotypes were observed, one for the sequences from *N. vison* and another for the sequences from *A. agrarius, M. meles* and *E. europaeus*. The overall variation between these haplotypes amounted to 1.4 % (3 nucleotides out of 219). In the case of ITS, amplification and sequencing generated four sequences of 1029–1042 bp. However, a 1014 bp alignment revealed that all the sequences from each host species were identical.

### Morphological analysis

**Fig. 1:**
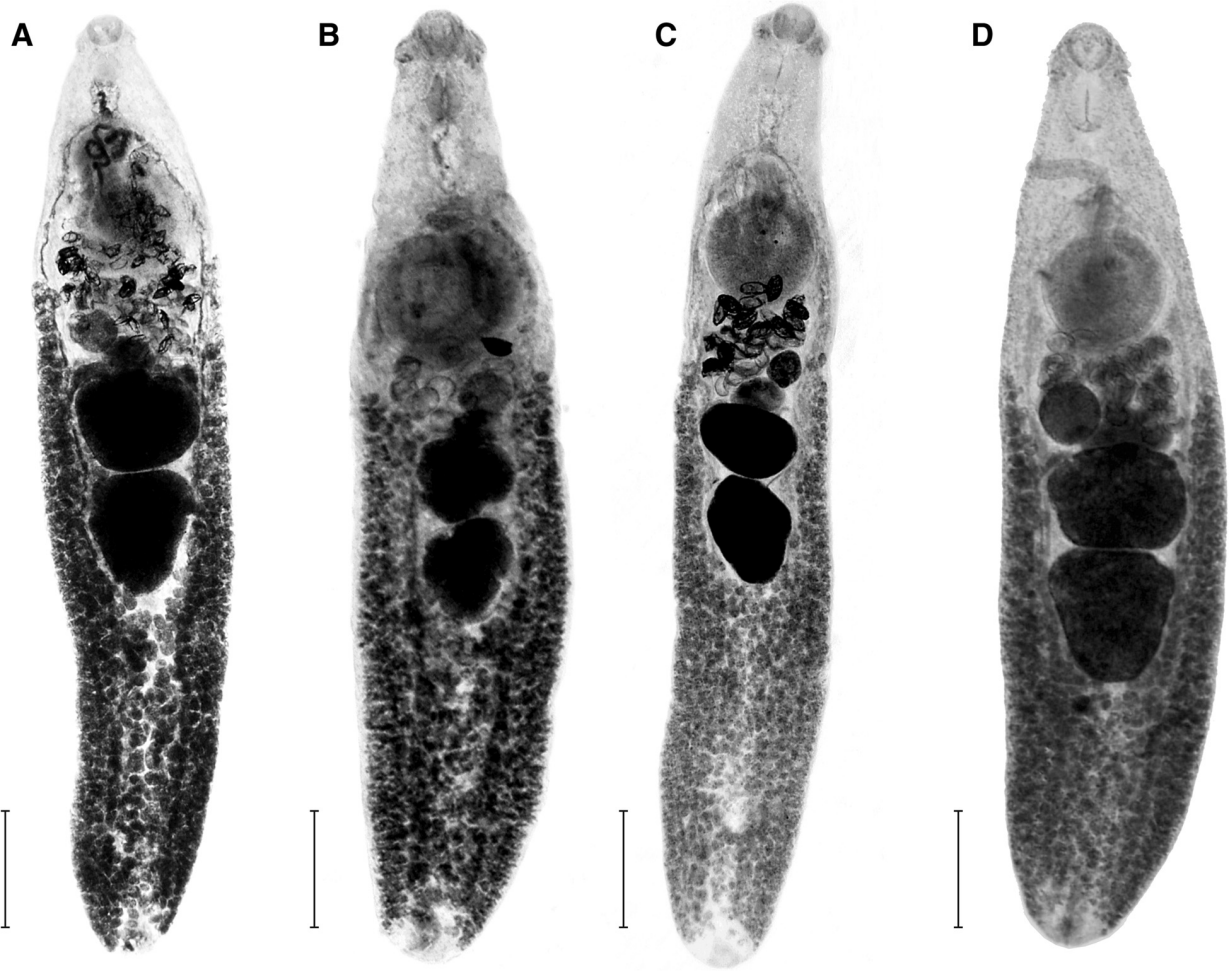
Morphology and body proportions of *Isthmiophora melis*. a – *M. meles,* scale bar – 1 mm; b – *N. vison,* scale bar – 0.5 mm; c – *E. europaeus,* scale bar – 0.6 mm; d – *A. agrarius,* scale bar – 0.3 mm

**Fig. 2:**
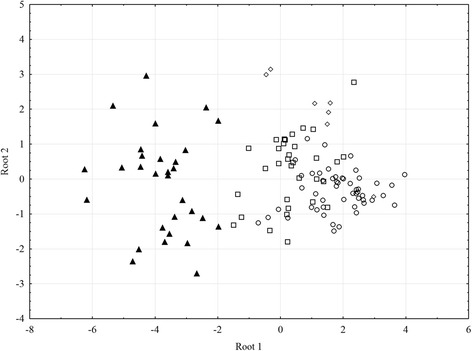
Results of canonical analysis of *Isthmiophora melis* obtained from four host species. Plot generated based on 6 variables measured in 148 specimens. Symbols denoting host species: circles – *N. vison,* squares – *E. europaeus*, diamonds – *M. meles,* black triangles – *A. agrarius*

The means, ranges and CV % values of *I. melis* from the four host species are shown in Table 2. The observed values of the analysed parameters demonstrate a high level of both intra- and between-host variation in the morphometric characters of the worms. The CV % values calculated for the variables expressed as ratios (BW, FO, H, T, U, GA/BA) were at the same level and did not show any statistically significant differences between the host species (F = 0.01; df = 3; *p =* 0.998). However, one-way analysis of variance (ANOVA) showed that host species played an important role in shaping the characteristics of *I. melis* (F = 20.3; df = 51; *p <* 0.001). The results of *post hoc* Tukey test showed that the differences were mostly associated with the trematodes from *A. agrarius* (Table 3). These specimens were characterised by a relatively smaller body size, higher values of maximum body width, expressed as proportion of body length (BW), a very short post-testicular region and therefore low values of the post-testicular region as a proportion of body length (T) (Fig. 1). The worms from *A. agrarius* displayed the highest values of relative gonad area/body area (GA/BA) (Fig. 1). In discriminant analysis (Table 4) the model was generated by the use of 6 variables. The chi-square test showed that the first three roots were required to separate *I. melis* among the four host species. The roots accounted for 91.5 % (Root 1), 96.6 % (Root 2) and 100 % (Root 3) of the overall variation. Root 1 separated the worms from *A. agrarius* based on the following variables, in order of descending importance: GA/BA, T, FO and U. The analysis also revealed that according to these criteria all the specimens of *I. melis* from *A. agrarius* were classified correctly (Table 5). Roots 2 and 3 separated trematodes from *M. meles* based on GA/BA and U, however this explained only 8.5 % of the variation. These results are also visible in the plot of canonical scores (Fig. 2) where *I. melis* from the striped field mouse are clearly separated from those isolated from the remaining hosts.

**Table 2:** Morphology of *Isthmiophora melis* from various hosts obtained in this study

	*Apodemus agrarius* (*N =* 37)			*Erinaceus europaeus* (*N =* 34)			*Neovison vison* (*N =* 64)			*Meles meles* (*N =* 13)		
	M	Range	CV %	M	Range	CV %	M	Range	CV %	M	Range	CV %
L	2,625	1,075–4,100	32	4,476	3,070–5,675	15	3,778	2,350–5,975	19	6,821	5,950–7,725	8
W	623	260–1,050	34	876	590–1,160	14	762	500–1,250	25	1,389	1,250–1,700	10
BA	1,411	219–2,967	57	3,113	1,422–4,344	24	2,356	1,021–5,863	45	7,468	5,838–10,309	16
BW	24	19–30	11	20	16–26	14	20	15–25	12	20	17–23	8
FB	665	315–950	26	904	630–1,030	10	754	530–1,090	16	1,083	960–1,210	8
FO	26	20–32	11	21	18–33	15	20	15–27	12	17	15–18	7
HB	1,558	550–2,750	39	2,997	1,900–3,925	19	2,487	1,680–4,050	21	4,613	4,225–4,940	6
H	58	31–69	12	67	54–83	7	66	47–80	8	71	69–72	2
PTR	692	293–1,225	33	1,640	1,010–2,060	18	1,486	1,000–2,300	19	2,570	2,100–3,025	11
T	27	20–32	11	37	31–46	8	39	34–46	7	38	35–41	5
OSA	22,566	8,247–53,066	42	35,747	22,687–43,352	12	26,897	15,386–57,227	33	57,060	46,163–70,650	14
VSA	127,434	24,732–277,910	49	266,453	79,133–468,454	29	185,377	54,091–515,036	51	508,892	424,077–653,635	15
ATA	118,293	36,287–250,592	53	152,747	37,994–237,463	32	93,766	35,448–248,379	57	447,761	110,391–671,666	35
PTA	129,210	28,339–296,907	53	167,942	37,994–270,948	28	104,482	39,740–259,541	60	475,179	186,560–671,665	31
OA	26,674	3,190–53,066	55	30,758	13,523–61,544	31	21,441	7,850–68,315	61	84,499	70,650–93,435	9
GA/BA	158	99–267	23	112	63–142	15	90	67–129	18	148	56–192	30
U	4	1–8	36	4	1–7	40	3	1–5	40	4	3–9	50
EL	131	120–140	5	129	120–140	5	121	115–125	4	127	115–140	8
EW	81	70–90	8	79	75–85	5	89	80–95	5	82	75–90	7

All measurements are expressed in micrometers; *M* mean, *Min* minimal value, *max* maximal value, *CV %* coefficient of variation; L-body length, W-maximum body width, BA-body area, BW-maximum body width as a proportion of body length, FB-forebody length, FO-forebody as a proportion of body-length, HB-hindbody length, H-hindbody as a proportion of body length, PTR-post-testicular region length, T-post-testicular region length as a proportion of body length, OSA-oral sucker area, VSA-ventral sucker area, ATA-anterior testis area, PTA-posterior testis area, OA-ovary area, GA/BA-gonad area/body area, U-ventral sucker to ovary distance as a proportion of body length, EL-egg length, EW-egg width

**Table 3:** Results of post hoc Tukey test of one-way analysis of variance (ANOVA)

	Aa/Ee	Aa/Nv	Aa/Mm	Ee/Nv	Ee/Mm	Nv/Mm
L	+	+	+	+	+	+
W	+	n/s	+	+	+	+
BA	+	+	+	+	+	+
BW	+	+	+	n/s	n/s	n/s
FB	+	n/s	+	+	+	+
FO	+	+	+	n/s	+	+
HB	+	+	+	+	+	+
H	+	+	+	n/s	n/s	n/s
PTR	+	+	+	n/s	+	+
T	+	+	+	+	n/s	n/s
OSA	+	n/s	+	+	+	+
VSA	+	n/s	+	+	+	+
ATA	n/s	n/s	+	+	+	+
PTA	n/s	n/s	+	+	+	+
OA	n/s	n/s	+	+	+	+
U	n/s	+	n/s	+	n/s	n/s
GA/BA	+	+	n/s	+	+	+

The data are presented pairwise for particular host species (Aa – *A. agrarius,* Ee – *E. europaeus,* Nv – *N. vison,* Mm – *M. meles*) and indicate statistical significance (+) or its lack (n/s)

**Table 4:** Summary of DFA; the table presents the full list of variables included in the analysis

Roots removed	Eigenvalue	Canonical R	Wilks’ lambda	Chi-square	df	*p*-value
0	5.207	0.916	0.104	259.857	18	< 0.001
1	0.298	0.479	0.648	49.909	10	< 0.001
2	0.189	0.399	0.841	19.931	4	< 0.001
	Wilks’ lambda	Partial lambda	*p*-value	Root 1	Root 2	Root 3
BW	0.108	0.966	0.274	−0.001	0.079	0.487
FO	0.132	0.794	< 0.001^*^	−0.501	−0.307	−0.698
H	0.111	0.944	0.093	0.282	0.201	0.079
T	0.123	0.854	< 0.001^*^	0.483	0.275	−0.460
U	0.127	0.824	< 0.001^*^	−0.334	0.230	−0.955
GA/BA	0.137	0.759	< 0.001^*^	−0.541	0.718	−0.063
Eigenvalue				5.207	0.298	0.190
Cumulative proportion				0.915	0.966	1.000

Statistically significant variables are marked with asterisk (^*^). Chi-square tests with successive roots removed are presented in the upper part of the table. Columns Root 1, Root 2 and Root 3 present standardized coefficients for canonical variables

**Table 5:** Classification efficiency of *Isthmiophora melis* from each host species

	% correct class.	*M. vison* (*p =* 0.422)	*E. europaeus* (*p =* 0.273)	*M. meles* (*p =* 0.057)	*A. agrarius* (*p =* 0.248)	Root 1	Root 2	Root 3
*M. vison*	92.2	47	4	0	0	1.825	−0.353	0.225
*E. europaeus*	72.8	8	24	1	0	0.364	0.256	−0.664
*M. meles*	71.4	2	0	5	0	1.132	1.919	0.769
*A. agrarius*	100	0	0	0	30	−3.768	−0.129	0.168
Total	87.6	57	28	6	30			

Columns Root 1, Root 2 and Root 3 reflecting the means of canonical values

## Discussion

The history of the genus *Isthmiophora* Luhe, 1909, especially in relation to the genus *Euparyphium* Dietz, 1909, is long and complicated, but both genera were established as valid by Kostadinova and Gibson [17]. The main characteristic features of *Isthmiophora* are: anterior position of the testes (proportion of length of post-testicular region to body length = 30–50 %), short forebody (FO = 10–20 %), presence of an armed cirrus, small head collar with 27 collar spines, varied size of dorsal spines (oral longer than aboral), short uterus and large eggs [17]. The life cycle of *Isthmiophora* includes lymnaeid snails, tadpoles and fish as intermediate hosts and carnivores as definitive hosts. Six species (*I. melis, I. hortensis* (= *Echinostoma hortense*)*, I. beaveri, I. citellicola, I. inermis, I. lukjanovi*) are currently regarded as valid [17]. *I. melis* is widespread in Europe, Asia and North America and uses more than 30 species of vertebrates as definitive hosts [19], including humans and rodents: *Apodemus agrarius, A. sylvaticus, Rattus norvegicus* and *Mus musculus* [22–24]. In Poland the species has been reported from fox, marten, badger, hedgehog and rodents [22, 24].

The specimens of *I. melis* from *A. agrarius* collected in Lower Silesia did not fully correspond to the description of *I. melis* [17], and two of the key features: forebody as a proportion of body length (FO) and post-testicular field as a proportion of body length (T), were distinct. According to Kostadinova [18], *Isthmiophora* possessed an intestinal bifurcation just anterior to the ventral sucker, the cirrus was armed and T = 30–50 % while *Euparyphium* was characterised by the intestinal bifurcation located halfway between the pharynx and the ventral sucker, unarmed cirrus and T = 20–30 %. The worms from *A. agrarius* had a short post-testicular field as a proportion of body length (T = 26.6 %), an armed cirrus and the intestinal bifurcation situated halfway between the pharynx and the ventral sucker. These features did not permit unambiguous identification of the trematodes as *Isthmiophora*. Additionally, Radev et al. [19] showed that in experimental infections of hamsters, specimens of *I. melis* still corresponded to the general description of the species, and the key features did not change significantly. Molecular identification, based on SSU and LSU of rDNA, of *I. melis* from the striped field mouse definitively confirmed their identity as *Isthmiophora*, while the less conservative markers (ITS1/ITS2 of rDNA and COI of mtDNA) pointed to a specific identity as *I. melis*. Additional specimens of *I. melis*, isolated from different hosts (*M. meles, N. vison* and *E. europaeus*), shared this molecular identity, with minimal (1.4 %) variation within the COI gene. Based on this molecular analysis, we must conclude that the echinostomatids collected from *A. agrarius* did represent *I. melis*. Nolan and Cribb [6] presented an extensive discussion of the role of ITS sequences in digenean taxonomy. Internal transcribed spacers in this group in general showed a small intraspecific variation, which was however sufficient to explore the validity of species boundaries in the group [6]. Morgan and Blair [12] also investigated the taxonomic position of eight 37-collar-spined echinostomatid species using ITS sequences and found that these spacer regions provided sufficient variation to distinguish 5 of the 8 nominal species examined, and the level of interspecific variation ranged between 1.1 % and 19.2 %. The remaining three species had identical ITS sequences and were indistinguishable. The same authors [13] also re-examined the same material using mitochondrial markers (CO1 and ND1), which allowed for unambiguous identification of the analysed material. Based on the reliability of the combination of nuclear and mitochondrial markers in these studies [6, 12, 13], we are confident that our specimens from *A. agrarius* do indeed represent *I. melis*.

The observed morphological variation must therefore be host-induced phenotypic variation, and its scale affects the diagnostic features at both generic and specific level. There is an extensive literature on the influence of population density on the echinostome morphology (e.g. the “crowding effect” of Fried and Freeborne [25, 26]), but we suspect an effect of host longevity as well. In general, the lifespan of carnivorous species is considerably longer than that of small rodents. The lifespan of *A. agrarius* in the wild approximates a few months (5–8) only, while, for example, the lifespan of *M. meles* is up to 15 years. The growth of body size and internal organs of trematodes is correlated at the initial phase. At a later period, when the gonads are fully developed, the body continues to grow with a simultaneous slower growth rate of gonads. For example, in experimental studies on the development of *E. revolutum,* Franco et al. [27] observed that gonads were fully developed 20–25 days post infection while full body size was only attained 55 days post infection. In our studies the highest values for the coefficient of relative gonad area to body area was observed in the trematodes from *A. agrarius*, indicating that the growth of the body had ceased; the values of this coefficient in the striped field mouse were almost identical as those observed in the badger – the type host for *I. melis*.

Genetic markers constitute a powerful tool in the studies on intraspecific variation in many taxa, including helminths, but the morphology still plays a crucial part in species descriptions [28]. However it is evident that morphology alone may not provide adequate taxonomic resolution and may lead to misidentifications. The phenotypic plasticity of helminths has been reported frequently in the literature, e.g. [29–32]. For example, Boyce et al. [30] explain the differences in the morphology of *Notocotylus malhamensis* Boyce et al. 2012 as a result of the presence of young adults in one of the hosts, i.e. the specimens of *N. malhamensis* in *Microtus agrestis* have not fully developed. The second possible reason of host-induced morphological differences in *N. malhamensis* is crowding effect. The wide host range of *I. melis* combined with the very different sizes of the hosts (e.g. badger vs. field striped mouse) makes the phenotypic plasticity even more spectacular. Our studies suggest that species identification is very subjective and, when descriptions of new species or even higher taxa are based on few specimens, misidentification is very likely. Thus the combination of morphology with molecular analysis and studies on life histories is most desirable when identifying parasites.

## Conclusions

The morphological traits of *Isthmiophora melis* are highly variable and host-dependent, and without molecular analysis they might lead to a description of a new species or even genus. Such a high level of intraspecific variation may be affected by the host’s longevity.
